# Effect of the Channel Length on the Transport Characteristics of Transistors Based on Boron-Doped Graphene Ribbons

**DOI:** 10.3390/ma11050667

**Published:** 2018-04-25

**Authors:** Paolo Marconcini, Alessandro Cresti, Stephan Roche

**Affiliations:** 1 Dipartimento di Ingegneria dell’Informazione, Università di Pisa, Via Girolamo Caruso 16, 56122 Pisa, Italy; 2 Univ. Grenoble Alpes, CNRS, Grenoble INP, IMEP-LaHC, F-38000 Grenoble, France; crestial@minatec.inpg.fr; 3 Catalan Institute of Nanoscience and Nanotechnology (ICN2), CSIC and The Barcelona Institute of Science and Technology, Campus UAB, 08193 Barcelona, Spain; stephan.roche@icn2.cat; 4 ICREA—Institució Catalana de Recerca i Estudis Avançats, 08010 Barcelona, Spain

**Keywords:** graphene ribbon, transistor, boron doping, channel length, transport, *I_ON_*/*I_OFF_* ratio, mobility gap

## Abstract

Substitutional boron doping of devices based on graphene ribbons gives rise to a unipolar behavior, a mobility gap, and an increase of the $I_{ON}/I_{OFF}$ ratio of the transistor. Here we study how this effect depends on the length of the doped channel. By means of self-consistent simulations based on a tight-binding description and a non-equilibrium Green’s function approach, we demonstrate a promising increase of the $I_{ON}/I_{OFF}$ ratio with the length of the channel, as a consequence of the different transport regimes in the ON and OFF states. Therefore, the adoption of doped ribbons with longer aspect ratios could represent a significant step toward graphene-based transistors with an improved switching behavior.

## 1. Introduction

Graphene is a two-dimensional honeycomb lattice of carbon atoms which was first systematically isolated by mechanical exfoliation from bulk graphite in 2004 [1], and is a material that presents many interesting properties [2,3]. For example, the envelope function equation of graphene formally coincides with the relativistic wave equation of massless spin-1/2 particles (the Dirac–Weyl equation) [4]. This has made it possible to experimentally observe relativistic phenomena such as Klein tunneling, Zitterbewegung, and anomalous quantum Hall effect, at the low energies that are typical of condensed matter physics [5,6,7,8]. Moreover, graphene is a one-atom-thick material that is light and flexible but with a large mechanical strength, it has very high electrical and thermal mobilities, and is transparent and impermeable. Many applications have been proposed for it, some of which are rapidly finding their way to the market [9,10,11].

From the point of view of electronic devices, the thinness, two-dimensional geometry, and high mobility of graphene have suggested its application as the conduction channel of scaled transistors. However, the absence of an energy gap in two-dimensional monolayer graphene has hampered the fabrication of graphene-based MOS (metal-oxide-semiconductor) transistors with a $I_{ON}/I_{OFF}$ ratio sufficiently high to allow their use for digital circuits [12] (the $I_{ON}/I_{OFF}$ ratio is the ratio between the current flowing through the device in its ON state and in its OFF state). The ambipolar behavior characteristic of pristine graphene represents a further drawback, since the possibility of using complementary devices would be desirable. To date, these limitations have limited the possible application of graphene transistors to analog electronics, particularly in the radiofrequency field [12,13]. Meanwhile, for the realization of graphene-based digital devices, alternative operating principles such as that of the tunnel-field-effect-transistor [14,15] have been proposed. However, many efforts have been focused on the improvement of the switching properties of graphene-based transistors. Even though the possibility of surpassing or even approaching the commutation properties of traditional transistors currently seems out of reach, the availability of well-operating digital graphene field-effect-transistors (FETs) would allow us to benefit from the excellent properties of this material. For example, it would make it possible to fabricate flexible, transparent, and low-cost all-graphene electronic circuits, high-speed devices, or devices in which also the mechanical, thermal, or optical properties of graphene could be exploited. Several methods have been proposed to induce an energy gap in graphene [16], such as transversal confinement [17,18], doping [19,20,21,22,23,24], strain [25,26], the introduction of an antidot lattice [27,28], functionalization [29,30], or the application of an electric field orthogonally to bilayer graphene [31].

A common substitutional dopant for graphene (and, more generally, carbon materials) is boron, an element of the III chemical group (and thus with only one valence electron less than carbon) that has a size comparable to carbon and presents a quite low ionization energy if substituted into the graphene lattice. In particular, in Reference [32] we have shown that the introduction of boron atoms in substitutional positions breaks the ambipolar behavior of graphene, thus giving rise to asymmetric device trans-characteristics, opens a mobility gap (i.e., an energy region with very low conductance), and increases the $I_{ON}/I_{OFF}$ ratio. While these effects become more pronounced when increasing the doping concentration, the values of the $I_{ON}/I_{OFF}$ ratio obtained in the devices examined in Reference [32] are still unsatisfactorily low. Here, starting from those results, we examine the possibility of improving the $I_{ON}/I_{OFF}$ ratio by increasing the length of the device channel. The analysis is performed by means of an atomistic simulation code in which the Poisson equation for the device electrostatics and a tight-binding-based non-equilibrium Green’s function (NEGF) transport calculation are self-consistently solved. The substitutional boron doping is mimicked through a proper distribution of fixed charges. To ensure the successful completion of the simulations, we adopt enhanced convergence schemes with variable convergence thresholds and a recursive scanning of the simulation domain. Our results show that the $I_{ON}/I_{OFF}$ ratio, the width of the mobility gap, and the asymmetry of the transport characteristics increase when longer ribbons are considered, due to the cumulative effect of the boron atoms distributed along the ribbon. We demonstrate that this improvement of the switching behavior is intimately related to the different transport regime in the ON and OFF states of the device. We conclude that the adoption of longer ribbons in graphene-based transistors should have a positive effect from this point of view, thus representing a further advancement in the direction of functional devices.

## 2. Method

The effect on transport of the boron impurities is strictly related to the details of the (short-range) atomistic potential around the boron dopants. Therefore, an envelope-function approach such as that of References [33,34,35,36] would not be accurate enough, and more computationally demanding atomistic models are needed. On the other hand, a complete ab-initio simulation of the considered device which includes thousands of atoms would not be numerically feasible. Therefore, for our simulations we adopted a tight-binding model, which is a more simplified approach but preserves atomistic details and is able to correctly reproduce ab-initio results. In our tight-binding scheme we have included only the $2p_{z}$ atomic orbitals of the carbon and boron atoms, which give rise to the delocalized π molecular orbitals and thus mainly determine the transport properties. In particular, we have used the NanoTCAD ViDES simulation code [37,38], which self-consistently solves the transport and electrostatic equations in the device.

Regarding the transport side of the simulation, the Schrödinger equation with open boundary conditions is solved within the Green’s function formalism [39]. More in detail, the Green’s function is obtained as

(1) $$G\left(E\right)={\left[E\;I-H-\Sigma_{S}-\Sigma_{D}\right]}^{-1}\;,$$

where *E* is the energy of the charge carriers, *I* the identity matrix, and *H* is the nearest-neighbor tight-binding Hamiltonian matrix including only the $2p_{z}$ orbitals. $\Sigma_{S}$ and $\Sigma_{D}$ are the self-energy matrices of the source and drain contacts, which enforce boundary conditions on the Schrödinger equation corresponding to the presence of Schottky contacts at the two ends. For the self-energies, the method described in Reference [40] is adopted, which mimics the effect of real metal contacts.

In order to determine the device electrostatics, the Poisson equation is solved (using the Newton–Raphson numerical technique) on a tridimensional domain including the device. We consider Neumann conditions (corresponding to constant—and in particular zero—electric field) at the boundaries of the solution domain, and Dirichlet conditions (constant potential) at the gates. In particular, the Poisson equation

(2) $$\nabla \left[\epsilon \left(\vec{r}\right)\nabla \phi \left(\vec{r}\right)\right]=-e\left[p\left(\vec{r}\right)-n\left(\vec{r}\right)\right]-\rho_{fix}\left(\vec{r}\right)$$

is solved over a rectilinear grid defined over the tridimensional domain. In this equation, ϵ is the dielectric constant, ϕ is the electrostatic potential, *e* is the elementary charge, *p* and *n* are the electron and hole concentrations, $\rho_{fix}$ is the density of fixed charge, and $\vec{r}$ is the position vector.

The transport and electrostatic equations are mutually connected. The Schrödinger equation depends on the potential energy $\phi (\vec{r})$ (i.e., on the solution of the Poisson equation), while the Poisson equation depends on the electron and hole concentrations, which can be obtained from the Green’s function of the system (i.e., from the solution of the transport calculation). To perform the simulation, we start from an initial guess potential, we perform the NEGF calculation, and then we solve the Poisson equation with the electron and hole concentrations obtained from the NEGF procedure. The new potential obtained from the Poisson equation is then passed to the NEGF module, and so on recursively until the norm of the difference between the potentials obtained in two successive cycles is less than a given threshold. In our approach, the free charge around each atom is uniformly spread in the cell of the rectilinear grid that contains the atom.

The current flowing through the device can be computed from the converged Green’s function through the Landauer equation:

(3) $$I=\frac{2e}{h}\int_{-\infty }^{+\infty }T\left(E\right)\left[f\left(E-E_{F_{S}}\right)-f\left(E-E_{F_{D}}\right)\right]\;dE\;.$$

here, *h* is Planck’s constant, *f* is the Fermi–Dirac occupation function, $E_{F_{S}}$ and $E_{F_{D}}$ are the Fermi energies of the source and drain, respectively, and the transmission coefficient T(E) is given by

(4) $$T=-\mathrm{Trace}\left[\left(\Sigma_{S}-\Sigma_{S}^{†}\right)G\left(\Sigma_{D}-\Sigma_{D}^{†}\right)G^{†}\right]\;.$$

The tight-binding parameters and the fixed charge distribution of our model have been calibrated [32] on ab-initio simulations for a simplified structure consisting of a graphene ribbon with a single substitutional boron atom at different positions along the ribbon transverse section. In Reference [32], we reported results obtained from ab-initio density functional theory (DFT) simulations, performed with the SIESTA code [41] with a local density approximation and a double-ζ basis set. From these simulations, it emerged that the quasi-bound states localized around the boron atom introduce resonant backscattering—and thus a conduction reduction—in the hole branch of the transmission spectrum. In the devices we simulated, this effect dominates over the electrostatic effect of the impurity [42,43,44,45,46]. The energy of the conductance dip depends on the position of the boron impurity with respect to the ribbon edges [43]. From a purely electrostatic point of view, the boron atom, being an electron acceptor, introduces a repulsive potential for electrons; this potential profile was also extracted from the ab-initio simulation. We compared [32] the transmission spectra and the potential profiles obtained from the DFT calculations with those obtained from the previously described self-consistent tight-binding calculation performed through the ViDES code. A very good agreement was obtained by using the tight-binding parameters typical of undoped graphene and a particular choice of fixed charges. In detail, all the onsite energies were assumed equal to zero. The transfer integral between nearest neighbor atoms was always taken equal to $t_{p}$ = −2.7 eV, except between atoms belonging to the edge dimers of armchair ribbons. In this last case, the transfer integral was set to $1.12\;t_{p}$, in order to account for the reduced interatomic distance at the edges, and to correctly reproduce the energy gap of the ribbon [18]. Regarding the distribution of fixed charges $\rho_{fix}\left(\vec{r}\right)$, the best fitting was obtained considering a fixed charge null at each carbon atom and equal to −e at each boron atom. This can be physically explained observing that the sum of the charge of the nucleus and of all the electrons that are not in the $2p_{z}$ orbitals is equal to +e for carbon atoms and to 0 for boron atoms. The total charge is the sum of this charge and of the charge of the electrons in the π orbitals (which derive from the $2p_{z}$ atomic orbitals—i.e., the only orbitals that are considered in the tight-binding calculation). Let us call LDOS the local density of π states for unit area and energy, $E_{F}$ the Fermi energy and $E_{i}$ the midgap energy. Then, the total charge contained in an area *S* of the ribbon (containing $N_{C}$ carbon atoms and $N_{B}$ boron atoms) is given by

(5) $$\begin{array}{c} +eN_{C}-e\int_{S}dS\;\int_{-\infty }^{+\infty }dE\;\mathrm{LDOS}\left(E\right)f\left(E-E_{F}\right)=\\ +eN_{C}-e\int_{S}dS\;\int_{-\infty }^{E_{i}}dE\;\mathrm{LDOS}\left(E\right)f\left(E-E_{F}\right)-e\int_{S}dS\;\int_{E_{i}}^{+\infty }dE\;\mathrm{LDOS}\left(E\right)f\left(E-E_{F}\right)\;. \end{array}$$

we can sum and subtract from Equation (5) the charge of the electrons that would fill all the states in the π valence bands; that is,

(6) $$-e\int_{S}dS\;\int_{-\infty }^{E_{i}}dE\;\mathrm{LDOS}\left(E\right)\;.$$

the total number of states in the π valence bands is half the total number of π states, which is $2(N_{C}+N_{B})$ because each atom contributes with two $2p_{z}$ states with different spin. Therefore, the value of Equation (6) is $-e(N_{C}+N_{B})$. Summing and subtracting this charge from Equation (5), we obtain that the total charge in *S* is given by

(7) $$\begin{array}{c} +eN_{C}-e\left(N_{C}+N_{B}\right)+e\int_{S}dS\;\int_{-\infty }^{E_{i}}dE\;\mathrm{LDOS}\left(E\right)\left[1-f\left(E-E_{F}\right)\right]+\\ \;-e\int_{S}dS\;\int_{E_{i}}^{+\infty }dE\;\mathrm{LDOS}\left(E\right)f\left(E-E_{F}\right)=-eN_{B}+e\int_{S}dS\;\left(p-n\right)\;. \end{array}$$

therefore, the fixed charge to consider in Equation (2) is given by a charge null at each carbon atom and equal to −e at each boron atom.

Here, we adopted this approach to study the transport behavior as a function of the channel length of MOS transistors where the channel is represented by a boron-doped graphene ribbon.

## 3. Numerical Results and Discussion

**Figure 1:**
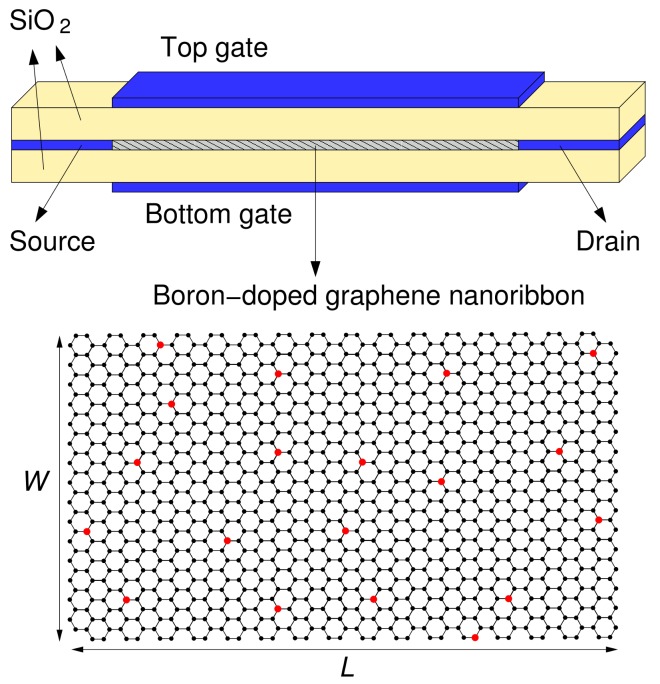
Sketch of the considered field-effect-transistors. The transmission channel is a boron-doped armchair graphene nanoribbon, represented at the bottom: black and red atoms are carbon and boron atoms, respectively, while the hydrogen atoms passivating the edges are not represented.

In particular, we have simulated a double gate FET (sketched in Figure 1). Its channel is an armchair graphene ribbon with width W= 3.81 nm (corresponding to 32 dimer lines) and length *L* variable between 10 nm and 70 nm. The channel is substitutionally doped with randomly located boron atoms, and connects the source and drain contacts. Two 1-nm-thick silicon oxide layers, located above and under the ribbon, separate it from the top gate and the bottom gate. The two gates are kept at the same potential and bias the transport channel. In our simulations, performed at room temperature, we applied a voltage $V_{DS}=$ 0.1 V between the source and the drain, and we computed the current $I_{D}$ flowing through the channel as a function of the voltage $V_{GS}$ applied between the gates and the source. In this way, we obtained the transfer characteristic $I_{D}\left(V_{GS}\right)$ of the device.

We solved the Poisson equation on a linear grid with a step of 0.1 nm inside the ribbon and progressively increasing towards 0.3 nm when approaching the domain boundaries.

Let us first consider the case of the undoped ribbon. When the gate voltage coincides with the average between the contact voltages (i.e., $V_{GS}=V_{DS}/2$), the energy of the charge carriers falls in the transition energy range between the valence and the conduction bands, where the density of states is minimum, and therefore the current is minimum. Instead, when increasing (decreasing) the gate voltage, the graphene band structure shifts towards lower (higher) energies. As a consequence, the energy of the charge carriers falls in the range of the conduction (valence) bands, where the electron (hole) concentration is large, and thus the current $I_{D}$ increases. This gives rise to a typical ambipolar transport behavior. In particular, in the case of the undoped ribbon, the transfer characteristic is symmetric with respect to $V_{GS}=V_{DS}/2$, as an effect of the symmetry of the dispersion relations with respect to the midgap energy.

In the presence of doping, the cumulative backscattering effect of the boron atoms breaks this symmetry and a unipolar behavior appears, together with a mobility gap. In our numerical simulations, we have considered several realizations of the random dopant spatial distribution. These have been used as a statistical ensemble for the extraction of average transistor characteristics.

**Figure 2:**
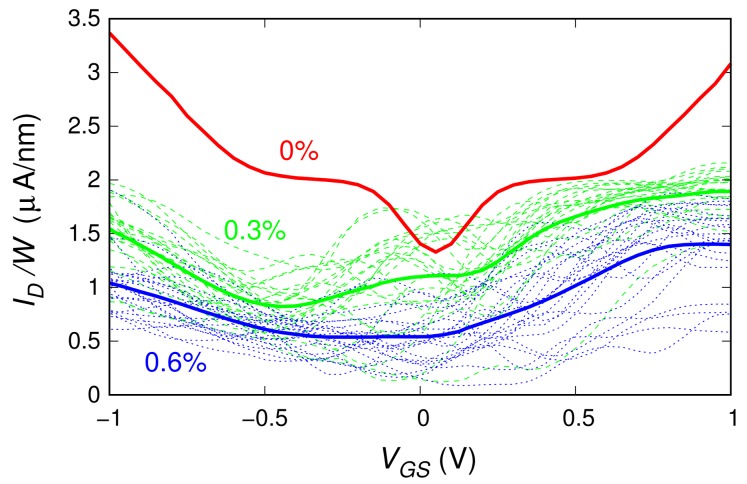
Transfer characteristics (obtained for $V_{DS}=0.1$ V) of the double-gate field-effect-transistor based on a 20-nm-long graphene ribbon with a boron concentration of 0% (undoped ribbon), 0.3%, and 0.6%. The thin lines (dashed for the 0.3% boron concentration, dotted for the 0.6% boron concentration) show the results for 23 individual realizations of the dopant distribution, while the thick lines represent the average over the ensemble. The current value was divided by the ribbon width W=3.81 nm in order to represent the current density.

Figure 2 shows the results reported in Reference [32] for a device based on a 20-nm-long graphene ribbon with a boron concentration equal to 0.3% and 0.6%, compared with those achieved for the undoped graphene ribbon. The boron concentration was defined as the ratio between the number of substitutional boron atoms and the total number of atoms within the channel. Numerical convergence problems precluded the simulation of ribbons with boron percentages larger than 0.6%. For each boron concentration, the dashed thin lines represent the characteristics obtained for the different random distributions of dopants, while the thick solid lines correspond to their average. When increasing the boron concentration, the hole and electron branches of the $I_{D}\left(V_{GS}\right)$ characteristic become more asymmetric, the voltage range for which we have low conduction through the device (and thus the mobility gap) widens, and the $I_{ON}/I_{OFF}$ ratio increases.

However, the values of the $I_{ON}/I_{OFF}$ ratio obtained with the 20-nm-long ribbon are still largely insufficient for a well-operating digital transistor. Indeed, a device with good switching properties should be characterized by a current flow in the OFF state much smaller than in the ON state. From this point of view, the adoption of longer doped graphene ribbons could improve the device behavior, due to the combined effect of a larger number of dopants. Therefore, our present analysis has focused on the dependence of the transport behavior on the length *L* of the boron-doped ribbon.

From the numerical point of view, the convergence of the self-consistent calculation is quite a difficult task, due to the presence of localized charges. In particular, this is particularly challenging for longer ribbons, which contain a larger number of dopants. To solve this problem, we adopted an enhanced convergence scheme with respect to Reference [32], based on a progressive approach to the final characteristic. In detail, we chose the starting potential profile for the self-consistent calculations in the following way. For the gate potential for which the convergence was found to be easier (in our simulations, $V_{GS}=0.5$ V) we used the flat band potential as a guess starting point. Then, the potential profile obtained at the end of the self-consistent simulation was used as a guess potential profile in the calculation performed for the successive gate potential value we considered. We repeated this procedure scanning the entire range of considered gate potentials a few times, alternatively moving for increasing and decreasing values of $V_{GS}$. At the same time, the error threshold adopted for the termination of the self-consistent calculations was progressively reduced to increase the accuracy of the solution. Finally, we considered that the convergence was achieved once the difference between the $I_{D}\left(V_{GS}\right)$ transfer characteristics obtained from successive scans of the domain became negligible.

**Figure 3:**
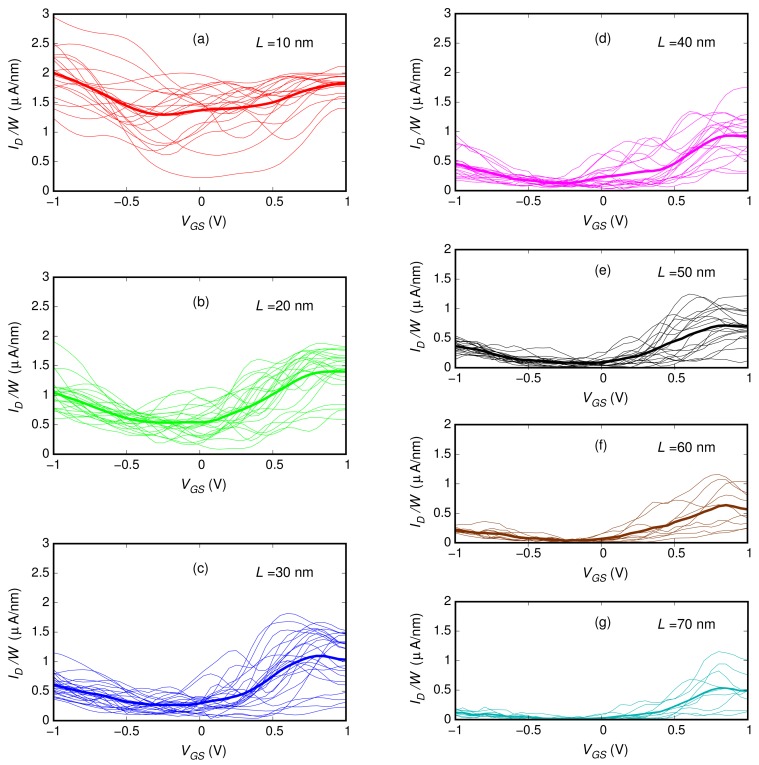
Transfer characteristics (obtained for $V_{DS}=0.1$ V) of the double-gate field-effect-transistor based on a graphene ribbon with a 0.6% boron concentration over a length of (**a**) 10 nm, (**b**) 20 nm, (**c**) 30 nm, (**d**) 40 nm, (**e**) 50 nm, (**f**) 60 nm, and (**g**) 70 nm . The thin lines correspond to the individual realizations of the dopant distribution, while the thick lines correspond to the average. The current value was divided by the ribbon width W=3.81 nm in order to represent the current density.

**Figure 4:**
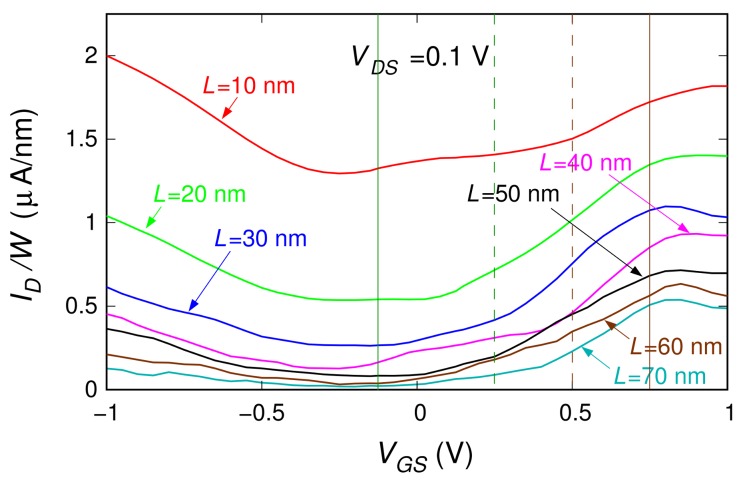
Average transfer characteristics (obtained for $V_{DS}=0.1$ V) of the double-gate field-effect-transistor based on a graphene ribbon with a 0.6% boron concentration over a length of 10, 20, 30, 40, 50, 60, and 70 nm. The current value was divided by the ribbon width W=3.81 nm in order to represent the current density. The four vertical lines identify the values of $V_{GS}$ for which the values plotted in Figure 6 were extracted.

**Figure 5:**
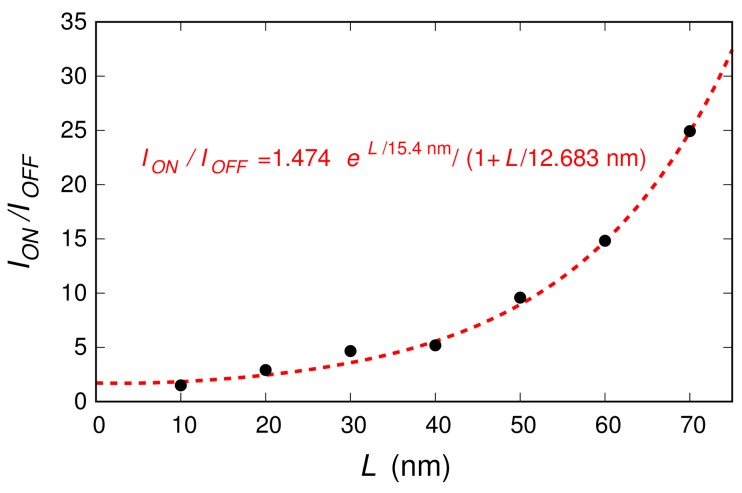
The black dots show the values of the $I_{ON}/I_{OFF}$ ratio obtained for the double-gate field-effect-transistor based on the graphene ribbon with a 0.6% boron concentration, for the seven considered ribbon lengths. The red dashed line represents the extrapolated analytical behavior.

We investigated 3.81-nm-wide ribbons with a 0.6% boron concentration (i.e., the highest percentage studied in the case of the 20-nm-long ribbon). We considered ribbons with lengths 10, 20, 30, 40, 50, 60, and 70 nm. For each length, we analyzed several different realizations of the boron dopant spatial distribution (20, 23, 24, 19, 18, 9, and 9 distribution realizations, respectively, for the seven considered lengths). In Figure 3, for each of the seven ribbon lengths, we report the sets of characteristics obtained for the different doping realizations (the dispersion of these curves is due to the low number of dopants). With a thicker line, we also plot the mean behavior achieved averaging, for each $V_{GS}$, the current obtained for the different boron distributions. These average behaviors are directly compared in Figure 4. In Figure 5, the black dots represent the values of the $I_{ON}/I_{OFF}$ ratio (obtained from these seven curves dividing the values of $I_{D}$ in the regions of high and low conduction) as a function of the ribbon length.

We observe that the increase of the graphene channel length has the positive effect of enhancing the $I_{ON}/I_{OFF}$ ratio. Moreover, it widens the OFF region of the transistor (i.e., the range of gate voltages for which the current flowing through the device is low). This can be explained by the fact that, for a given concentration of boron atoms, longer channels contain a larger number of dopants. The combined scattering action of the dopants on the charge carriers flowing through the device gives rise to the observed positive effect. At the same time, we observe an overall decrease of the current profile. However, this detrimental effect could be compensated by operating several transistors in parallel.

**Figure 6:**
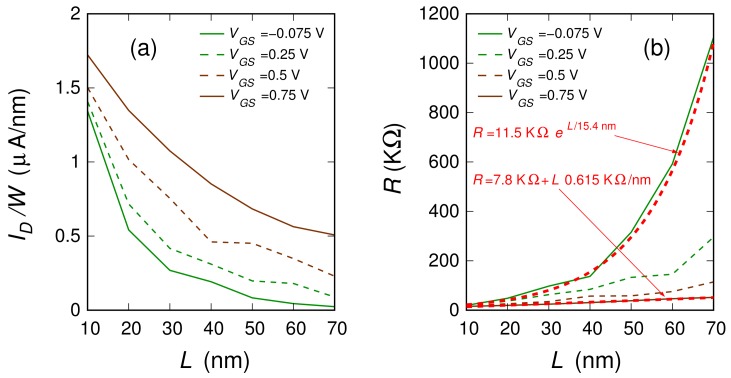
(**a**) Current density $I_{D}/W$ (with W=3.81 nm) and (**b**) Channel resistance $R=V_{DS}/I_{D}$ as a function of the length *L* for the four values of the voltage $V_{GS}$ identified by the vertical lines in Figure 4. In panel (**b**), the dashed red curves indicate the fit of the channel resistance as a function of the channel length for $V_{GS}=-0.075$ V and $V_{GS}=0.75$ V, which we take as representative of the OFF state and ON state, respectively, based on the analytical formulas for localized and diffusive transport regimes.

In order to better analyze our numerical results, Figure 6a reports the behavior of the current as a function of the length *L* for the four values of the voltage $V_{GS}$ identified with vertical lines in Figure 4. In Figure 6b, we show the corresponding values of the channel resistance $R=V_{DS}/I_{D}$ as a function of the length *L*. When the device is in its OFF state, the resistance *R* increases exponentially with the length *L*, thus suggesting that the device is working in a strong localization transport regime. Instead, when moving towards the ON regime, the resistance dependence on the channel length becomes linear. The transport in the ON state is therefore diffusive. Only when further increasing the channel length, the transport regime in the ON state is expected to become localized, with an exponential suppression of the current on a length scale much larger than that in the OFF state. In further detail, let us assume the behaviors for $V_{GS}=-0.075$ V and $V_{GS}=0.75$ V (where, approximately, the minimum and the maximum current regimes are reached in all cases) as representative of the OFF state and of the ON state, respectively. Then, a good fit of the dependence of the device resistance as a function of the channel length is given by the relations

(8) $$R_{OFF}=\left(11.5\;K\Omega \right)\times \exp\left(\frac{L}{15.4\;\mathrm{nm}}\right)$$

in the OFF regime, and

(9) $$R_{ON}=\left(7.8\;K\Omega \right)+\left(0.615\;\frac{K\Omega }{\mathrm{nm}}\right)L$$

in the ON regime. Since $R=V_{DS}/I_{DS}$ and $V_{DS}$ is the same in the two regimes, the $I_{ON}/I_{OFF}$ ratio should be reasonably fitted by the relation:

(10) $$\frac{I_{ON}}{I_{OFF}}=\frac{R_{OFF}}{R_{ON}}=\frac{1.474\times \exp\left(\frac{L}{15.4\;\mathrm{nm}}\right)}{1+\frac{L}{12.683\;\mathrm{nm}}}.$$

This is verified in Figure 5, where we plot this expression as a dashed red curve. Indeed, it appears to fit well the seven black dots, which represent the values of the $I_{ON}/I_{OFF}$ ratio that we have previously computed for the considered ribbon lengths.

Equation (10) allows us to extrapolate some considerations for longer ribbons, for which a complete numerical treatment would be too computationally challenging. Indeed, following Equation (10), further increasing the channel length *L* should lead to a nearly-exponential enhancement of the $I_{ON}/I_{OFF}$ ratio. Therefore, the adoption of longer graphene ribbons with a sufficiently high boron doping concentration could be usefully exploited in order to achieve graphene-based devices with an improved switching behavior and asymmetric electron–hole transport characteristics.

## 4. Conclusions

We numerically analyzed the transport characteristics, as a function of the channel length, of MOS transistors based on graphene ribbons randomly doped with substitutional boron atoms. The study was carried out through self-consistent quantum simulations based on a tight-binding atomistic model, validated through comparison with ab-initio DFT calculations. The numerical complexity of the calculations, increasing with the length of ribbons, required the adoption of enhanced convergence schemes. From the results of our simulations, performed for ribbon lengths ranging from 10 nm to 70 nm, we were able to observe a strongly localized transport in the OFF operating region and a diffusive transport in the ON operating region. This made it possible to extrapolate an analytical, nearly exponential relationship between the $I_{ON}/I_{OFF}$ ratio and the channel length. In addition, by increasing the length of the channel we observed an enhanced electron–hole asymmetry in the $I_{D}\left(V_{GS}\right)$ characteristics and a widening of the voltage range corresponding to the mobility gap. The results of our simulations suggest that graphene-based transistors should strongly benefit—from the point of view of both the $I_{ON}/I_{OFF}$ ratio and the device unipolarity—from an increase of the length of the boron-doped channel. Therefore, this solution could be exploited to improve the switching performances of graphene-based devices.
